# Explainable stock prices prediction from financial news articles using sentiment analysis

**DOI:** 10.7717/peerj-cs.340

**Published:** 2021-01-28

**Authors:** Shilpa Gite, Hrituja Khatavkar, Ketan Kotecha, Shilpi Srivastava, Priyam Maheshwari, Neerav Pandey

**Affiliations:** 1 Symbiosis Institute of Technology, Symbiosis International (Deemed University), Pune, Maharashtra, India; 2 Symbiosis Center for Applied Artificial Intelligence (SCAAI), Symbiosis International (Deemed University), Pune, Maharashtra, India

**Keywords:** Long Short-Term Memory (LSTM), Explainable AI(XAI), Stock price prediction, Deep Learning

## Abstract

The stock market is very complex and volatile. It is impacted by positive and negative sentiments which are based on media releases. The scope of the stock price analysis relies upon ability to recognise the stock movements. It is based on technical fundamentals and understanding the hidden trends which the market follows. Stock price prediction has consistently been an extremely dynamic field of exploration and research work. However, arriving at the ideal degree of precision is still an enticing challenge. In this paper, we are proposing a combined effort of using efficient machine learning techniques coupled with a deep learning technique—Long Short Term Memory (LSTM)—to use them to predict the stock prices with a high level of accuracy. Sentiments derived by users from news headlines have a tremendous effect on the buying and selling patterns of the traders as they easily get influenced by what they read. Hence, fusing one more dimension of sentiments along with technical analysis should improve the prediction accuracy. LSTM networks have proved to be a very useful tool to learn and predict temporal data having long term dependencies. In our work, the LSTM model uses historical stock data along with sentiments from news items to create a better predictive model.

## Introduction

Stock market investment/trading can be very tricky and stressful but rewarding if predicted correctly. It has been an object of study for the past many decades and is a complex task because of the large number of parameters, disordered information, and dynamism. Several technical indicators and sources of information affect the stock prices, but due to the substantial amount of data present, it becomes difficult to predict the prices. However, with the advancement in technology, particularly in processing large chunks of temporal data, the field is continuously improving to achieve better prediction accuracy.

There is a famous hypothesis in finance called the Efficient Market Hypothesis, which states that asset prices cannot entirely depend on obsolete information and market prices react to new information, for example, financial news articles, social media blogs, etc (Ţiţan, 2015). These sources change the sentiments of the investor and the traders. With the advancement in Artificial Intelligence, information coming from both financial time series, which captures sentiments and other technical analysis data, can be fused to predict stock prices.

In this paper, we suggest a technique involving LSTM and the interpretability power of Explainable AI (XAI) for visual representations to provide a definite outline that helps them anticipate their future stock prices. The data we are using is the National Stock Exchange (NSE) and the news headlines aggregated from Pulse (Pulse by Zerodha, 2020). Pulse has aggregated 210,000+ Indian finance news headlines from various news websites like Business Standard, The Hindu Business, Reuter, and many other news websites.

## State-of-the-art Techniques

Cho et al. (2014) proved that the Recurrent Neural Network (RNN) is a powerful model for processing context information from textual data. However, to tackle long-term dependencies, the variant of RNN, LSTM, has proved to be very effective in handling complex tasks for text processing and language modeling on temporal data (Sherstinsky, 2020). We propose using LSTM for news classification for sentiments, employing the interactions of words during the compositional process. LSTM incorporates a memory cell, which is a unit of computation that supersedes the traditional deep learning neurons in the network (Moghar & Hamiche, 2020; Egeli, Ozturan & Badur, 2003). To understand the behavior of the proposed model, we also intend to make our model explainable. XAI aims to develop a collection of machine learning techniques that produce more explainable models (Doran, Schulz & Besold, 2017). Using XAI techniques, we wish to provide knowledge about the prediction made by the model so that the user can get insights for future trading/investment strategies. The model can be interpreted by visual tools, which can help us to consider the biases in the model before making the final decision.

Kalyani, Bharathi & Rao (2016) in their research, using supervised machine learning for classification of news headlines and additional text mining techniques to examine news polarity. The news articles with its polarity score and text converted to tf-idf vector space are fed to the classifier. Three different classification algorithms (Support Vector Machines “SVM”, Naïve Bayes and Random Forest) are implemented to investigate and enhance classification accuracy. Results of all three algorithms are compared based on precision, recall, accuracy, and other model evaluation techniques. When evaluating the results of all classifiers, the SVM classifier performs satisfactorily for unknown data. The Random Forest also showed better results when compared to the Naïve Bayes algorithm. Finally, the relationship between news articles and stock price data are plotted.

Nayak, Pai & Pai (2016) used the historical data from 2003 obtained from Yahoo Finance and used two models to predict the stock trend. One model was built for the prediction of daily stock by considering all the data available daily. The second model that was built was for monthly prediction of stocks, and it considered data available every month. Also, two different datasets were used for each model. A historical price dataset was used for the Daily Prediction model and historical data from 2003 obtained from Yahoo finance is used for a monthly prediction model. The dataset was modeled using various models like boosted decision tree, logistic regression, and support vector machine. Up to 70% accuracy was observed using the Support Vector Machine.

Vargas, Lima & Evsuko (2017) proposed a Recurrent Convolutional Neural network model. It predicts intraday movements of the S&P 500 record. The model data sources financial news headlines from the day past of the forecast day and utilizes a few specialized indicators which are extracted from the primary target. Every news is processed through a two-step procedure—initially, a word2vec model to create a vector to display each word, and afterward they implement the mean (average) of all the word vectors of the same title. The RCNN model uses deep learning models: CNN and RNN. The CNN model is utilized to separate rule base data from the text while the RNN-LSTM model is utilized to get the context information and to interpret the stock information attributes for forecast purposes.

Yoo, Kim & Jan (2005) analyzed and assessed a portion of the current ML techniques for stock exchange prediction. After comparing various models like multivariate regression, Neural Networks, Support Vector Machines, and Case-Based Reasoning models, they inferred that Neural Networks offer the capacity to predict market trends accurately when contrasted with different procedures. SVMs and Case-Based Reasoning are famous for predicting stock costs due to their simplicity of use and implementation.

### LSTM

LSTM (Long Short-Term Memory) is an improved form of RNN. LSTM models avoid the problems encountered by RNN. Hochreiter & Schmidhuber (1997) introduced LSTMs that make use of memory cells that can either forget unnecessary information or store information for more extended periods. LSTMs are explicitly modeled to handle tasks involving historical texts and are also able to educate themselves on long term dependencies. With the help of memory cells, they are capable of educating themselves. LSTMs have a chain-like structure making it easier to pass on information. The information is passed on as a state of the cell from one memory cell to another. The output of the network is modified by the state of these cells.

**Figure 1:**
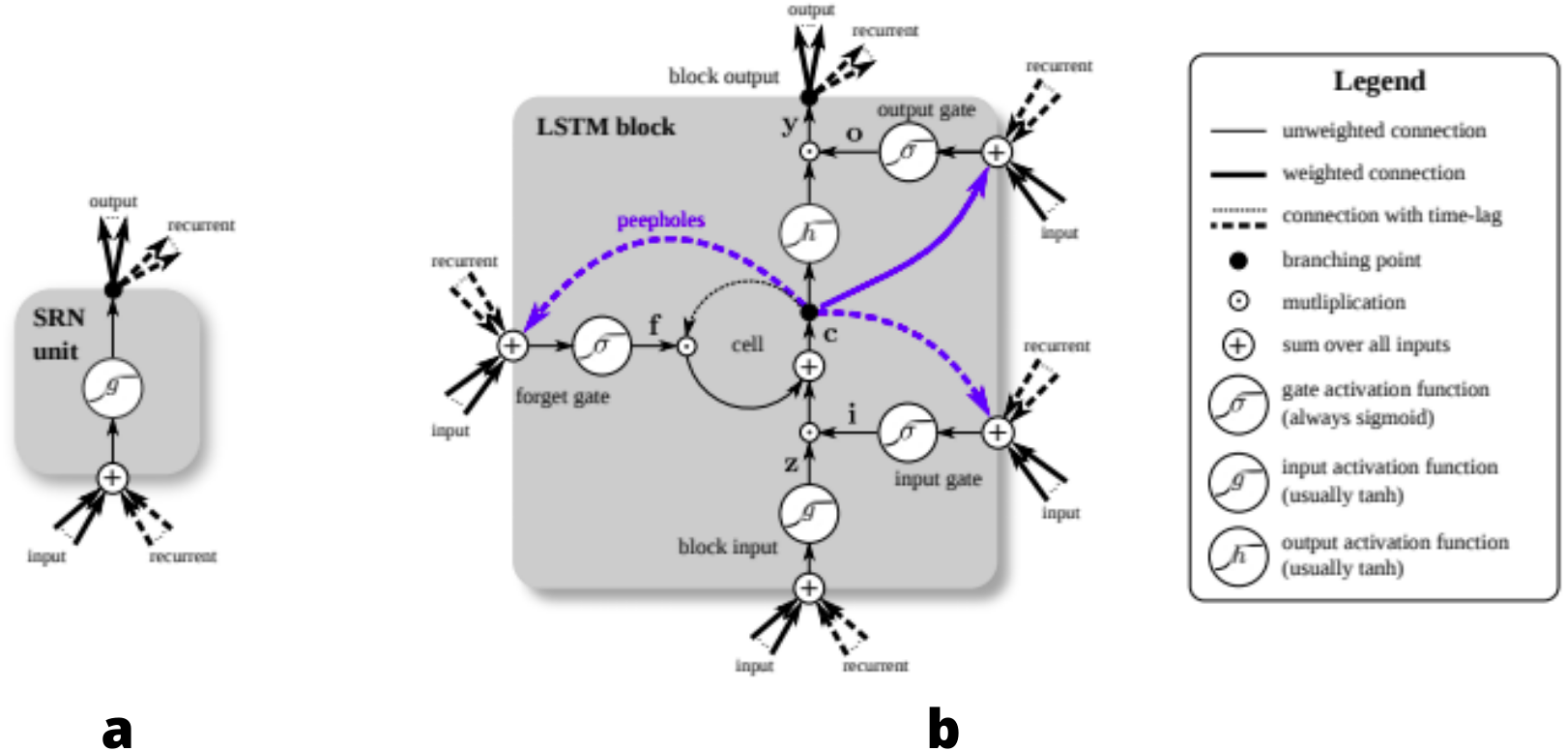
Detailed schematic of the Simple Recurrent Network (SRN) unit (A) and a Long Short-Term Memory block (B) as used in the hidden layers of a recurrent neural network.

The architecture of LSTM allows for constant error flow with the help of constant, self-connected units (Hochreiter & Schmidhuber, 1997). This flow of error and states is propagated with the help of the three gates: input gate, output gate and forget gate, that each LSTM memory cell block is composed of. Input gates modulate the amount of new information received by a cell, forget gates determine what amount of information from the previous cell is passed on to the current cell; they determine what information is relevant and what information needs to be forgotten (Kim & Won, 2018). Given below in Fig. 1A is the structure of Simple Recurrent Network (SRN). As compared to SRN, LSTM-Cell given in Fig. 1B is different and has multiple gates.

LSTM model has two passes:

• Forward Pass, and
• Backward Pass

#### Forward Propagation

In neural networks, the storage and calculation of intermediate results in the sequence from the input layer to the output layer are called Forward Propagation. In this propagation, we work incrementally through the mechanics of a deep network with one hidden layer. It consists of the processing of weighted inputs through the activation function. Forward propagation is done to get a value. We then compare this computed value with the real value to further compute the error. Then, in backward propagation, we calculate the derivative of the error with respect to the weights. Then we subtract the value obtained from the value of the weight. The final step in a forward pass (Vargas, Lima & Evsuko, 2017) is to the comparison between the predicted and the expected output.

Forward propagation is calculated using the following steps:

(1.1) \begin{eqnarray*}{Z}^{[i]}& ={w}^{[i]}{a}^{[i-1]}{+b}^{[i-1]}\end{eqnarray*}

(1.2) \begin{eqnarray*}{g}^{[i]}(Z)& =\sigma ({Z}^{[i]}).\end{eqnarray*}

#### Backpropagation

Backpropagation (Vargas, Lima & Evsuko, 2017) is analogous to calculating the delta rule for a multilayer feedforward network. Backpropagation is the method to calculate the gradient of neural network parameters. The main aim of backpropagation is to minimize the cost function by weights and biases of the network. The amount of adjustment to be made is determined by the gradients of the cost function for the respective parameters. Assuming we have the following functions:

(1.3) \begin{eqnarray*}Q& =f(P)\end{eqnarray*}

(1.4) \begin{eqnarray*}R& =g(Q)\end{eqnarray*}

(1.5) \begin{eqnarray*}g(Q)& =g\circ f(P).\end{eqnarray*}

Where, the input and the output P, Q, R are tensors of arbitrary shapes. By using the chain rule, we can compute the derivative of

R wrt P:

(1.6) \begin{eqnarray*}\mathrm{dR}/\mathrm{dP}=\mathrm{dot}(\mathrm{dR}/\mathrm{dQ},\mathrm{dQ}/\mathrm{dP}).\end{eqnarray*}

### A Forward Pass

Let x^t^ be the input for time t, N be the quantity of LSTM blocks, M is the number of inputs. We then get the subsequent weights for LSTM layer:

•Input weights: W_z_, W_i_ , W_f_ , W_o_ ∈ R^*N*×*M*^

•Recurrent weights: R_z_, R_i_ , R_f_ , R_o_ ∈ R^N×N^

•Peephole weights: p_i_ , p_f_ , p_o_ ∈ R^N^

•Bias weights: b_z_, b_i_ , b_f_ , b_o_ ∈ R^N^

Then the vector formulas for a vanilla LSTM layer forward pass can be written as:

(1.7) \begin{eqnarray*}{\underline{z}}^{\mathrm{t}}& ={\mathrm{W}}_{\mathrm{ z}}{x}^{\mathrm{t}}+{\mathrm{R}}_{\mathrm{ z}}{y}^{\mathrm{t}-1}+\mathrm{bz}\end{eqnarray*}

block input (1.8) \begin{eqnarray*}{\mathrm{z}}^{\mathrm{t}}& =g({\underline{z}}^{\mathrm{t}})\end{eqnarray*}

(1.9) \begin{eqnarray*}{\underline{i}}^{\mathrm{t}}& ={\mathrm{W}}_{\mathrm{ i}}{x}^{\mathrm{t}}+{\mathrm{R}}_{\mathrm{ i}}{y}^{\mathrm{t}-1}+{p}_{\mathrm{ i}}\odot {c}^{\mathrm{t}-1}+{\mathrm{b}}_{\mathrm{ i}}\end{eqnarray*}

input gate (1.10) \begin{eqnarray*}{\underline{i}}^{\mathrm{t}}& =\sigma ({\underline{i}}^{\mathrm{t}})\end{eqnarray*}

(1.11) \begin{eqnarray*}{\underline{f}}^{\mathrm{t}}& ={\mathrm{W}}_{\mathrm{ f}}{x}^{\mathrm{t}}+{\mathrm{R}}_{\mathrm{ f}}{y}^{\mathrm{t}-1}+{p}_{\mathrm{ f}}\odot {c}^{\mathrm{t}-1}+{\mathrm{b}}_{\mathrm{ f}}\end{eqnarray*}

forget gate (1.12) \begin{eqnarray*}{f}^{\mathrm{t}}& =\sigma ({\underline{f}}^{\mathrm{t}})\end{eqnarray*}

cell (1.13) \begin{eqnarray*}{\underline{f}}^{\mathrm{t}}& ={\mathrm{W}}_{\mathrm{ f}}{x}^{\mathrm{t}}+{\mathrm{R}}_{\mathrm{ f}}{y}^{\mathrm{t}-1}+{p}_{\mathrm{ f}}\odot {c}^{\mathrm{t}-1}+{\mathrm{b}}_{\mathrm{ f}}\end{eqnarray*}

(1.14) \begin{eqnarray*}{\mathrm{f}}^{\mathrm{t}}& =\sigma ({\underline{f}}^{\mathrm{t}})\end{eqnarray*}

(1.15) \begin{eqnarray*}{\mathrm{c}}^{\mathrm{t}}& ={z}^{\mathrm{t}}\odot {i}^{\mathrm{t}}+{\mathrm{c}}^{\mathrm{t}-1}\odot {\mathrm{f}}^{\mathrm{t}}\end{eqnarray*}

(1.16) \begin{eqnarray*}{\underline{o}}^{\mathrm{t}}& ={\mathrm{W}}_{\mathrm{ o}}{\mathrm{x}}^{\mathrm{t}}+{\mathrm{R}}_{\mathrm{ o}}{\mathrm{y}}^{\mathrm{t}-1}+{\mathrm{p}}_{\mathrm{ o}}\odot {\mathrm{c}}^{\mathrm{t}}+{\mathrm{b}}_{\mathrm{ o}}\end{eqnarray*}

(1.17) \begin{eqnarray*}{\mathrm{o}}^{\mathrm{t}}& =\sigma ({\underline{o}}^{\mathrm{t}})\end{eqnarray*}

Output gate (1.18) \begin{eqnarray*}{\mathrm{y}}^{\mathrm{t}}& =\mathrm{h}({\mathrm{c}}^{\mathrm{t}})\odot {\mathrm{o}}^{\mathrm{t}}\end{eqnarray*}

where *σ*, g and h are pointwise non-linear activation functions. The logistic sigmoid (*σ*(*x*) = 1∕1 + *e* ^−*x*^) is employed as an activation function and also the tangent hyperbole (g(x) = h(x) = tanh(x)) is typically used because the block input and output activation function. Pointwise multiplication of 2 vectors is denoted by ⊙ (Greff et al., 2015) .

### B. Backpropagation

Calculation of deltas in LSTM Block:

(1.19) \begin{eqnarray*}\delta {\mathrm{y}}^{\mathrm{t}}& ={\Delta }^{\mathrm{t}}+{\mathrm{R}}_{\mathrm{ z}}^{\mathrm{T}}{\delta }_{\mathrm{ z}}^{\mathrm{t}+1}+{\mathrm{R}}_{\mathrm{ i}}^{\mathrm{T}}{\delta }_{\mathrm{ i}}^{\mathrm{t}+1}+{\mathrm{R}}_{\mathrm{ f}}^{\mathrm{T}}{\delta }_{\mathrm{ f}}^{\mathrm{t}+1}+{\mathrm{R}}_{\mathrm{ o}}^{\mathrm{T}}{\delta }_{\mathrm{ o}}^{\mathrm{t+1}}\end{eqnarray*}

(1.20) \begin{eqnarray*}\delta {\underline{o}}^{\mathrm{t}}& =\delta {y}^{\mathrm{t}}\odot \mathrm{h}({c}^{\mathrm{t}}){\sigma }^{{^{\prime}}}({\underline{o}}^{\mathrm{t}})\end{eqnarray*}

(1.21) \begin{eqnarray*}\delta {\mathrm{c}}^{\mathrm{t}}& =\delta {\mathrm{y}}^{\mathrm{t}}\odot {\mathrm{o}}^{\mathrm{t}}\odot {\mathrm{h}}^{{^{\prime}}}({\mathrm{c}}^{\mathrm{t}})+{\mathrm{p}}_{\mathrm{ o}}\odot \delta {\underline{o}}^{\mathrm{t}}+{\mathrm{p}}_{\mathrm{ i}}\odot \delta {\underline{i}}^{\mathrm{t+1}}+{\mathrm{p}}_{\mathrm{ f}}\odot \delta {\underline{f}}^{\mathrm{t+1}}+\delta {\mathrm{c}}^{\mathrm{t}+1}\odot {\mathrm{f}}^{\mathrm{t}+1}.\end{eqnarray*}

Here, Δt is the vector of deltas delegated from the layer above. If E is the loss function, it corresponds to ∂E ∂yt , but not counting the recurrent dependencies (Pathmind, Inc., 2020). Then:

(1.22) \begin{eqnarray*}\delta {\underline{f}}^{\mathrm{t}}=\delta {\mathrm{c}}^{\mathrm{t}}\odot {\mathrm{c}}^{\mathrm{t}-1}\odot {\sigma }^{{^{\prime}}}({\underline{f}}^{\mathrm{t}})\end{eqnarray*}

### Why LSTM?

LSTM networks various state cells. These short and long-term memory cells rely on the state of these cells. These memory cells act as an aide for the model to remember historical context as predictions made by the network are influenced by past experiences of inputs to the network. This helps us make better predictions. LSTM networks tend to keep the context of information fed by inputs by integrating a loop that allows information to flow, in one direction i.e from one step to the following.

### Explainable AI (XAI)

We want our model to output not only the prediction part but also the explanation as to why the prediction turned out that way. If our machine makes a prediction, why should the user trust the prediction made by the machine? Today, machine learning and Artificial Intelligence(AI) are exploited to make decisions in many fields like medical, finance, and sports. There are cases where machines aren’t 100% accurate. So, the user should blindly trust the choice of the machine? How can the user trust AI systems that derive inferences on probable unfair grounds? To solve the problem of trust between the user and Artificial Intelligence, XAI (Saffar, 2019) can be employed. It gives us the reasoning for a prediction made by the model. Mainly XAI is employed to resolve the black box problem. This ”black box” phase is interpreted by XAI and explained to the users. The user cannot completely depend on the model without a clear understanding of the model and the way the output is achieved. XAI provides a clear understanding of how the model achieved a certain prediction or result. XAI gives a human-understandable explanation for the prediction. Current models enable interpretation but leave it to the user to apply their knowledge, bias and understanding.

## Methodology

Given below is a systematic representation of how the data served to the system, how the model is trained and tested. And how XAI works to interpret the model.

### System design

**Figure 2:**
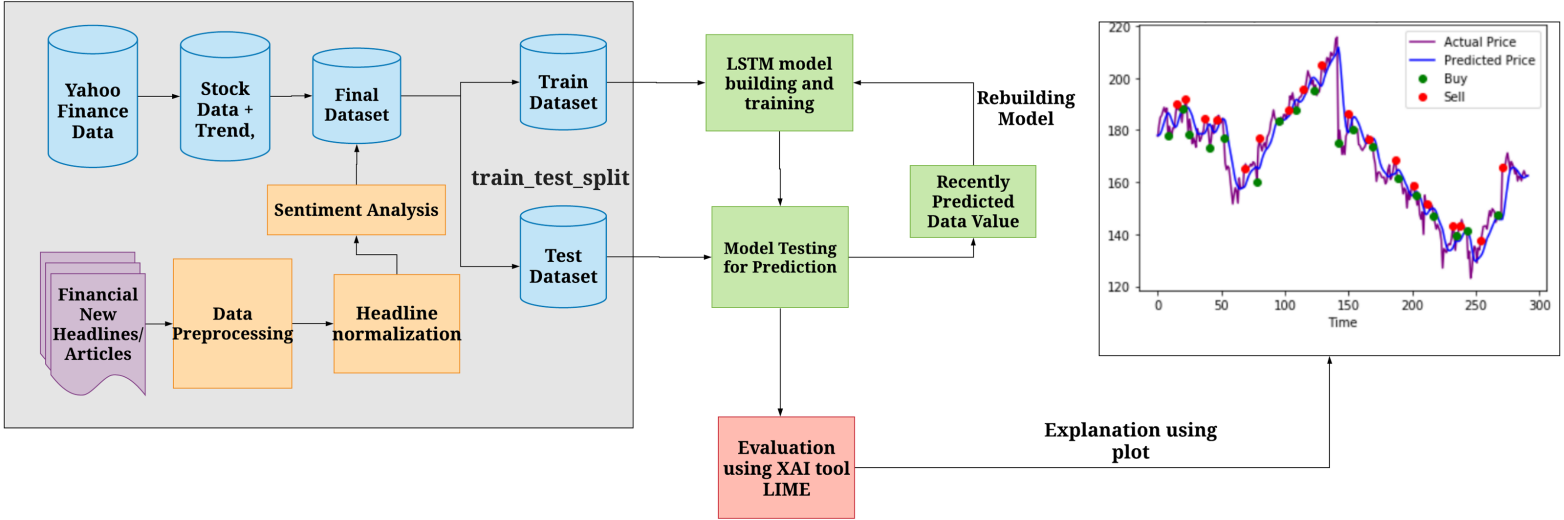
System Architecture of Stock Market Prediction using LSTM and XAI. Shows how the data is initially processed, divided into train and test set to build further predictions and evaluate the biases using XAI.

Figure 2 explains the overall architecture of the system. We preprocess the News Headlines Dataset by performing tokenization, removing stop words, and embedding it. The headlines are then normalized and sentiment analysis is performed on it to comprehend the sentiment behind each sentiment, i.e whether a particular headline has a positive or negative sentiment associated with it. We then use the preprocessed headlines to classify them. We classify them to analyze whether they produce a positive sentiment or a negative sentiment. Later, this headline dataset, along with the preprocessed Yahoo Finance dataset, is combined to form the final dataset. Then the dataset is divided into train and test dataset. The training dataset is used to build the LSTM model. The test dataset to verify the results obtained after training. Finally, XAI is implemented using the LIME tool that is used to interpret the model and understand the biases involved in the dataset.

#### Algorithm

The algorithm is divided into prediction of the opening price using LSTM-CNN and XAI for interpreting the model. Given below is the algorithm.

### Prediction

**Table:** 

Input: processed news headlines
Instantiate ReduceLRPlateau()
Instantiate ModelCheckpoint()
Instantiate EarlyStopping()
Truncate and pad input sequence
while epoch r: 1 ->R
For batch_size b: 1->B
Design the model by adding:
Sequential layer to LSTM model
embedding Layer
Conv1D() and MaxPooling1D
LSTM()
Dense layer
Fit the model
Evaluate the model
Save the model
Use XAI to interpret the model
Output: Table with Predicted opening values

### XAI

**Table:** 

Input: Processed headlines
Instantiate LimeTextExplainer()
Instantiate explain_instance() with appropriate hyperparameters(text, pipeline.predict_proba, number of features)
Create an ordered dictionary of words and weights
Plot a bar-plot with appropriate axes
Output: Plot of biases of words (weights) vs words

### Procedure

#### Selecting the prediction model

Based on the evaluation done by Ghosh et al. (2019) comparing the various models, including simple linear regression such as ARIMA, AR, ARMA, or nonlinear models such as ANN, ARCH, RNN, LSTM it was concluded that LSTM could achieve higher accuracy as compared to other models. Stock market prediction requires a nonlinear dynamic system; LSTM can train themselves with nonlinear input–output pairs. Moreover, combining qualitative factors like international events, financial news, News headlines’ sentiments, etc. can achieve much higher accuracy.

#### Selecting datasets

A dataset of financial news headlines is obtained from Pulse. Pulse is a news aggregator and it aggregates Financial news from various sources thus providing less biased dataset. This dataset is combined with a Yahoo Finance dataset (Yahoo Finance, 2020). Since our main focus was on Indian Stock Market prices (Hiransha et al., 2018), we extracted news headlines from the dataset accordingly. Moreover, an indication of changes in the stocks (Zhang, Fuehres & Gloor, 2011) for BSE and NSE was included. The values represented the stock market prices of the day the news headline was published and of the day after. The dataset was split into 80% for training, 10% for validation, and 10% for testing. The dataset used in this project has 19,736 data entries and has 3 parameters: New Headlines, Website and the Timestamp.

#### Data preprocessing

**Figure 3:**
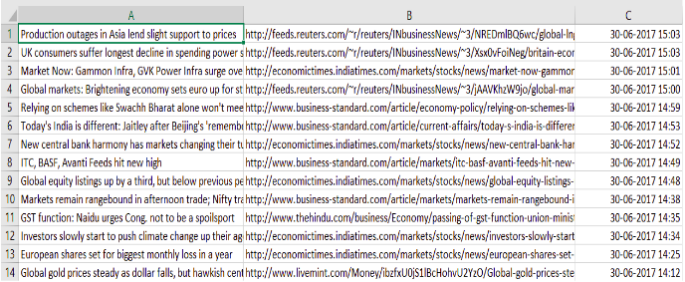
Dataset before cleaning. The data collected through various sources consists of unwanted and redundant values that need to be processed.

The data obtained is raw and unprocessed. To perform any computation, processing the data is necessary. For instance, the null data should be removed, and the trivial values should be removed, etc. Given below in the Fig. 3 is the raw dataset obtained from the sources.

This is what the Financial News dataset looked like before preprocessing. Table 1 shows the features neccessary for our model and we clean the dataset to obtain only these features.

**Table 1:** Features of Clean data. Final columns that are used in the dataset that is necessary for the model building and model evaluation.

Column A	Column B	Column C
News headline	Website	Timestamp

**Figure 4:**
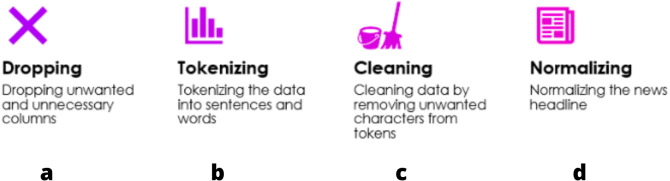
Steps in data preprocessing. (A) Dropping: to include only the necessary data, unwanted columns/rows must be dropped; (B) Tokenization helps in increasing the speed of computation and efficiency; (C) cleaning is required after tokenization since some words are not necessary for the evaluation; (D) normalizing: by bringing all values in one range, it is easier to compare various values and evaluate the model.

Steps involved in Data Preprocessing are represented in Fig. 4:

As we are first training the LSTM model only for BSE-SENSEX and NSE-NIFTY, we train it on news headlines involving SENSEX and NIFTY specific news only. For this, we search for headlines that contain the word: “Sensex” (case - independent).

**Figure 5:**
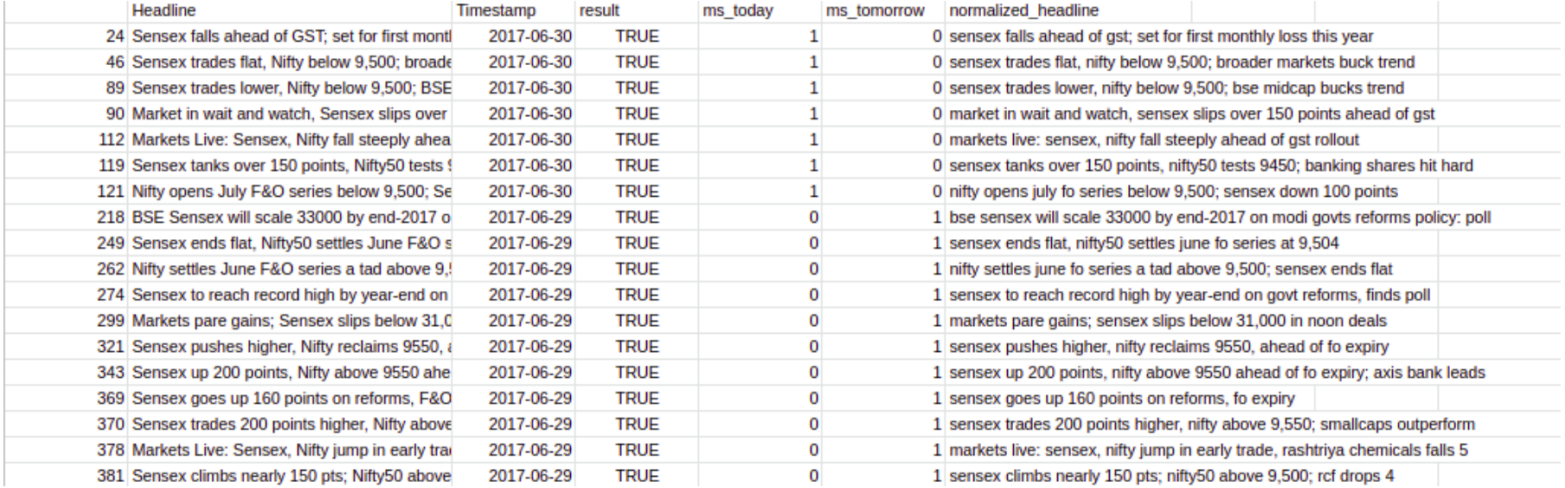
Dataset after cleaning. Clean dataset, after preprocessing helps in an efficient model building and model evaluation.

After running the code on our cleaned dataset we get this result as shown in Fig. 5.

#### Sentiment analysis for stock market news headlines

**Figure 6:**
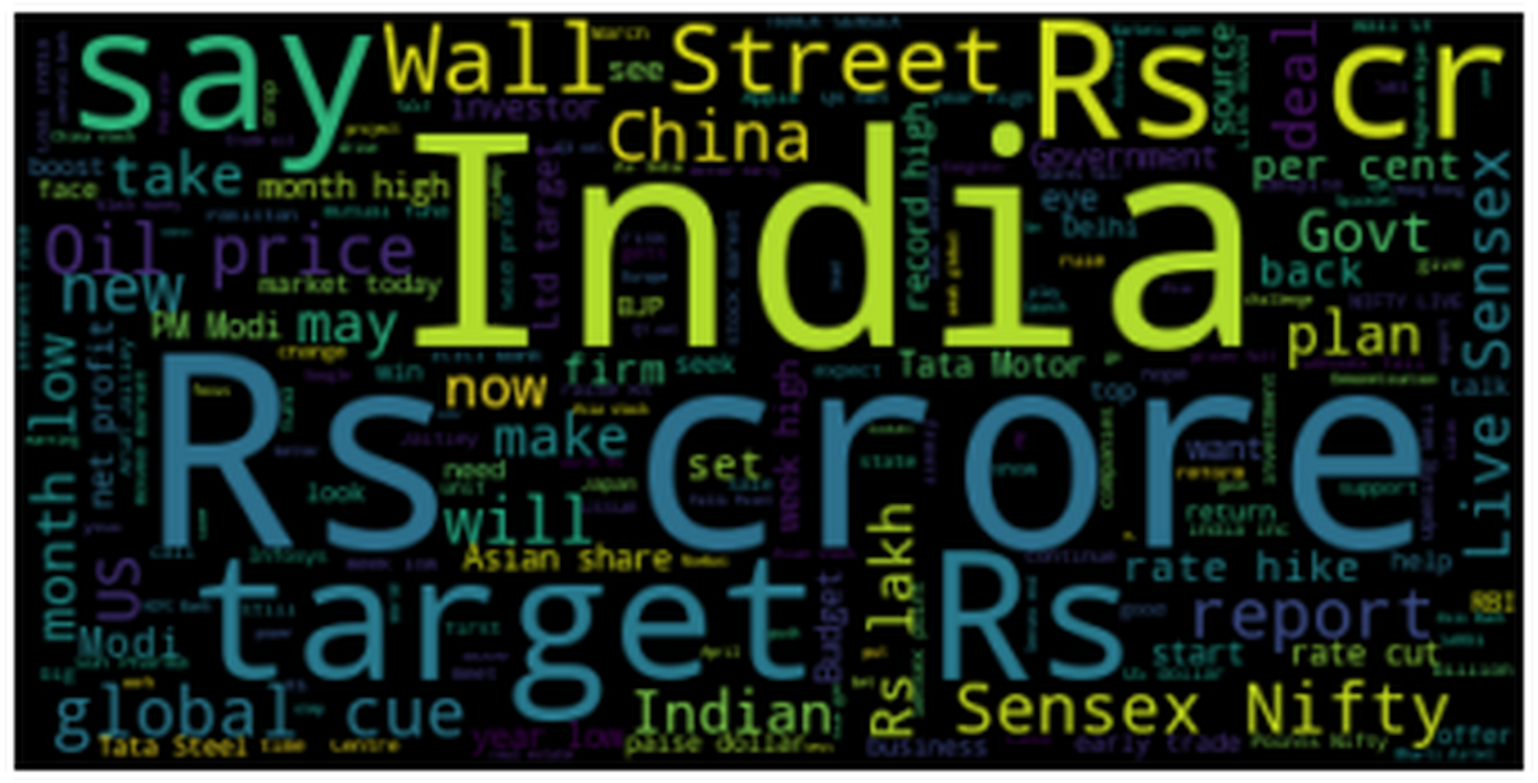
Top 150 commonly used words in a news headline by using WordCloud. The WordCloud generated from the data gives the overview of which words in the headline impact stocks.

To be able to predict the stock market (Patel et al., 2014), we must understand the market’s sentiment correctly. Negative news will lead to a fall in the price of the stock and positive news will lead to rising in the price of the stock. The most commonly used words in a news headline will give rise to an instant trigger of emotions in a particular person’s mind. Hence we made a word cloud of the top 150 words commonly occurring in a news headline from our dataset as depicted below in Fig. 6.

**Figure 7:**
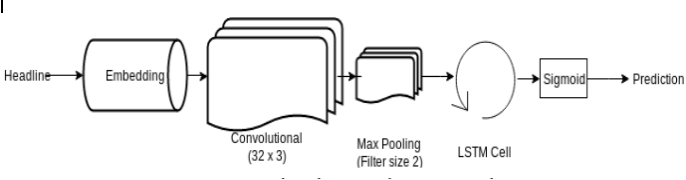
Network Architecture of LSTM Network. The network architecture of the LSTM network depicts how the processed headlines are embedded and passed through a CNN Layer and further through an LSTM cell to output the prediction.

LSTM network is a type of RNN that preserves its state. (Olah, 2020) LSTM (Selvin et al., 2017) has already provided satisfactory results in the area of text data applications such as machine translation and NLP (Bird, Klein & Loper, 2016). The headlines are a type of text data; for that reason, it was a rational decision to apply this type of network to our data. Figure 7 shows the LSTM network architecture used in our project.

#### Input

News features from different online news sites have been scraped and used as input for our application. Then the headlines are cleaned, and the remaining characters are converted to lowercase to overcome identical double words with different capitalization. Word embedding is dealt with by changing over the words to their proper index using the top 2000 words in the corpus; the rest of the words that are not found in the top 2000 words corpus are set to zero. The maximum size of the word embeddings is 100 and zero-padded for smaller headlines.

#### LSTM - CNN model for news headlines

1. Initially, we have created an instance of ReduceLROnPlateau, which reduces the learning rate when a metric has stopped improving accuracy.
2. Secondly, we have a ModelCheckpoint callback. It is used for training the model using model.fit() to save a model or weights at some interval, which can be loaded later to continue the training from the state saved.
3. An instance of EarlyStopping is created. This stops training the model when a particular metric has stopped improving. Then the preprocessed data is loaded and only the top n words are kept, and the rest are zero. Also, split it into the train dataset and test dataset. The data is then truncated and we pad the input sequence (X_train and X_test). A split of 70-30 is done for the train and test.
4. Finally, we create a model.
  (a) The first layer is constructed using the sequential network model of the Keras framework. A Sequential model works good enough for vanilla stack layers. Each layer has one input tensor and one output tensor exactly.
  (b) The next layer, i.e., an Embedding layer, is added. Here, the text data is encoded first. In an embedding, words are defined by dense vectors. Each vector serves the projection of the word into a continuous vector space.
  (c) Then a MaxPooling1D layer is added. A pooling layer is an elementary constituent unit of CNN. The main function of the pooling layer is to sequentially minimize the spatial orientation size and the number of parameters and hence reduce the computation required over the network. The pooling layer operates separately for each feature map.

**Figure 8:**
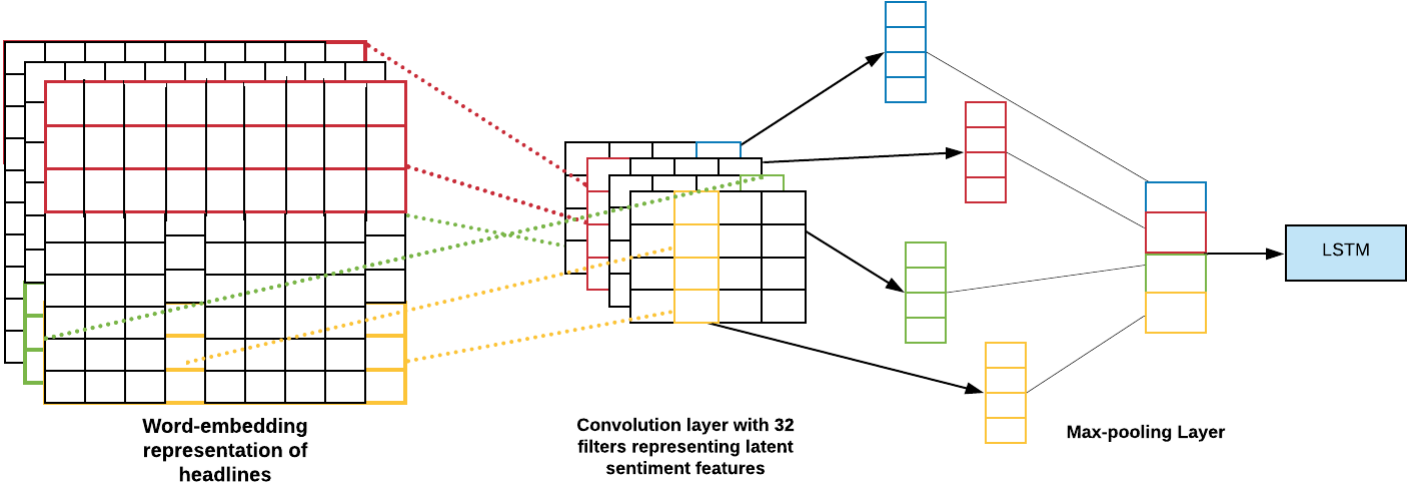
CNN MaxPooling1D. The MaxPooliing1D layer minimizes the spatial orientation size. The layer works separately on each feature.

Out of all the pooling layers, the one best suited for our model was max pooling. It is depicted as follows: It takes the maximum value over the window defined by pool_size and hence downsamples the input representation. In our model, the pool_size is 2. Figure 8 visualises the working of CNN MaxPooling1D.

Next, we then construct an LSTM layer of 100 units, as our inputs’ maximum review length is 100. We then add a Dense layer having the activation function of sigmoid as this is our final layer. We also are using an adam optimizer with a learning rate of 1e^−3^. The model is finally compiled and then we save the model. We then print the model summary and the function is called for today and tomorrow parameters.

#### LSTM model for OHLC dataset

The OHLC dataset consists of Open, High, Low and Close stock prices of a particular company for each period. For this model, we have initially loaded the dataset and split it into test and train data. This dataset does not require much preprocessing as the data and operation over it is very straightforward. Once data is loaded we use MinMaxScaler() which transforms features by scaling each feature to a given range. The fit_transform method is applied to the instance scl of MinMaxScaler() to tokenize the text and output, each dimension corresponding to the frequency of token found in the text. The data is split into input ‘X’ and output ‘y’. It is collected by splitting ‘i’ past days as input X and ‘j” coming days as Y.

The fit_transform method is applied to the instance scl of MinMaxScaler() to tokenize the text and output. Then we build the model with the first layer constructed using the Sequential model and the next 2 hidden layers using LSTM Finally, a Dense() layer is used for constructing the output.

#### Hyperparameters, loss function and optimizer

A hyperparameter is a configuration whose value cannot be determined by the dataset. It needs trial experiments where one can manually test which hyperparameter suits best for the model.Once fine-tuned the model is trained better.

Optimizers are algorithms for altering the attributes as weights and learning rate in order to reduce the loss. Table 2 represents the hyperparameters and their values used in the experiment.

#### LIME

**Figure 9:**
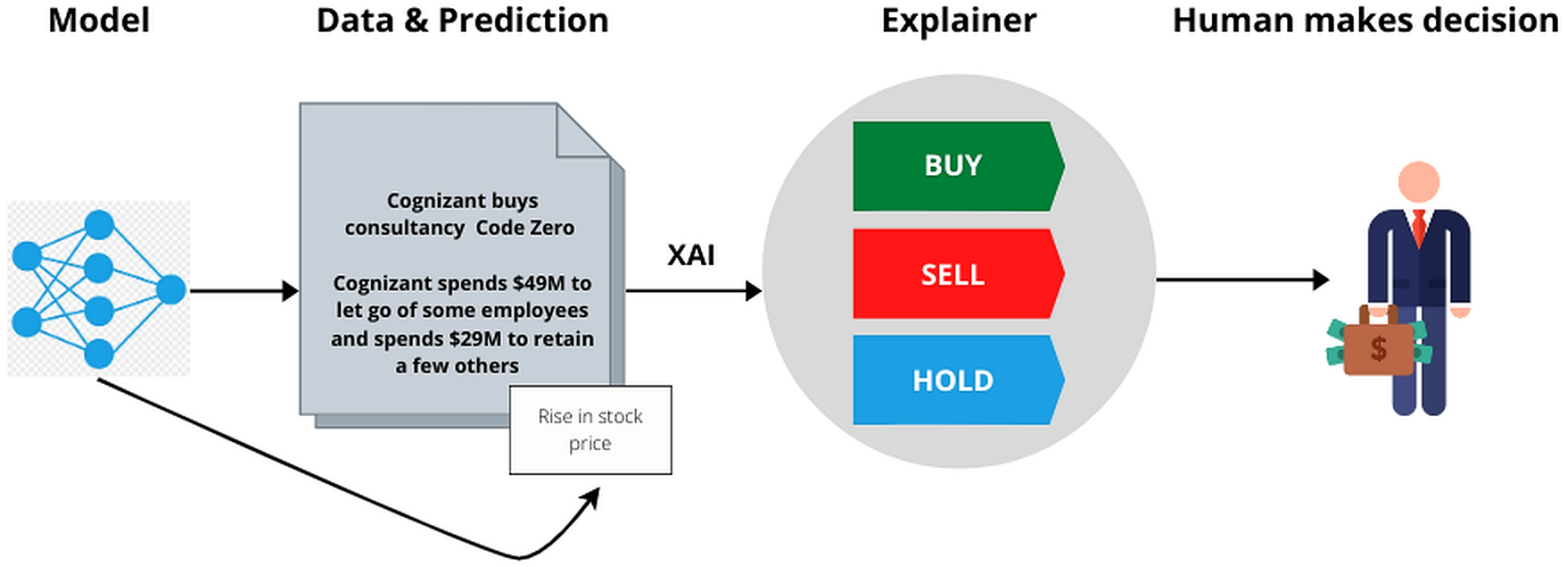
Working of XAI. The mode generates the prediction, which along with the data, is run through the XAI tool. The XAI tool explains the data based on the biases to facilitate better decision making.

As represented in Fig. 9, after training the model and obtaining predictions the explainer interpretes the model so that the user can make appropriate judgements. After studying various XAI (Khaleghi, 2019; Ribeiro, Singh & Guestrin, 2016) tools like LIME, What-If (Wexler et al., 2019), DeepLift (Shrikumar et al., 2016), Shapely (Messalas, Christos & Yannis, 2019), we realized the one which will determine the Transparency of our model in the best possible way was LIME.

**Table 2:** Hyperparameters, loss function and optimizer and their values used along with detailed comments.

**Title**	**Value**	**Comments**
Loss function	Binary cross-entropy	Since the type of classification performed is binary
Optimizer	Adam optimization	Used to enhance the network
Learning rate	0.001	Set after trials
Decay	0.1	Validation loss does not ameliorate significantly any more for 5 epochs, there will be decay.

According to the study, the What-if Tool is an interactive visual tool to help the user comprehend the datasets and better understand the output of TensorFlow models. To analyze the models deployed. However, it is well suited for XGBoost and SciKit Learn Models.

DeepLift is a tool that collates the activation of a neuron to its ’reference points’ and assigns contribution scores accordingly. A single backward pass is used to compute scores efficiently. DeepLift reveals dependencies that are otherwise missed by other methods giving separate consideration for positive and negative contributions.

LIME, short for Local Interpretable Model-Agnostic Explanations (Ribeiro, Singh & Guestrin, 2016), is a tool that is used to explain what the machine learning model is performing. It is applied to the models which perform behaviour but we do not know how it works.

Machine learning models (Patel et al., 2015) can be explained using plots, for example, linear regression, decision trees; these are some of the classifiers that can be explained using plots (It can only give an explanation for the global, but it cannot give an explanation of any local observation). But there are some significant data with more complexities and more dimensions that cannot be explained using plots. One of the examples is the neural network; it cannot be explained using plots.

By using LIME, we can understand what the model does, which features it picks on to create or develop prediction. LIME is model-agnostic; it means that it can be used for any black-box model that we know today or can be developed in the future. LIME tool is local; it means it is observation specific; it explains every single observation that you have in a dataset. If a model has a high accuracy, can we trust the model without having interpretability? The answer is no; we cannot trust it because many models have noises that can predict the output right, but the way the model has predicted will contain some faults which are not good for a long term process. So, interpretability is important for building trust with the model.

### Performance measure

#### Validation loss

For neural networks we consider the loss to be negative log-likelihood for classification and residual sum of squares for regression. Hence the primary aim of our learning model is to decrease the value of the loss function as we tune its parameters by changing weights through different optimization methods. The value of loss suggests how well a certain model runs for each optimization iteration. Preferably, after each iteration, we would expect a decrease in the value of the loss. Some points should be kept in mind while we observe the decrease in loss value. For example, one may face the problem of overfitting wherein the model memorizes the training dataset examples and becomes less effective for the test dataset. Over-fitting also occurs when you do not employ a regularization or normalization. We may have a very intricate model, or the number of data points N is very low.

#### Validation accuracy

We calculate the accuracy of a model once the model is completely trained. This means that the parameters are fixed, and no further model learning will be taking place. Then the test dataset is then inputted into the model, and the number of errors the model makes is reestablished after comparing it with the target values. Finally, the percentage of misclassified data is calculated. Let us understand this with an example. The model’s accuracy is 96.2% means that out of 1,000 test samples, the model classifies 962 of samples correctly.

## Results and Discussion

### Result

Table 3 represents the results generated after performing the experiments.

**Table 3:** Result generated. The LSTM-CNN model results using the New Headlines dataset and its comparison with the result generated by the LSTM model using the OHLC dataset. The result depicts the accuracy and loss of the two models.

Model	Dataset	No of Epochs	Accuracy	Loss
LSTM-CNN	News Headlines	15	74.76%	0.1693
LSTM	OHLC	10	88.73%	0.1733

In LSTM-CNN the input was the data that we get from preprocessing. The data was then combined with a dataset of the news headlines and the prices. The model which we trained was of the news headlines of Sensex.

There are a total of 100 epochs to be run in the training of the model. But it stops at the 15th epoch; this is due to avoiding making the model overfitting. The accuracy of the model in a total of 15 epochs is 74.76%. The Loss was 0.1693.

**Figure 10:**
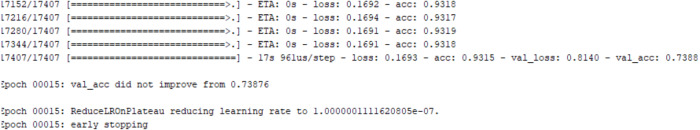
Loss LSTM -CNN model for News Headlines. The loss values for the LSTM-CNN model showing that the model was processed till Epoch No. 16, and there was an Early Stopping to avoid overfitting.

As we see in Fig. 10:

Epoch 00015:val_acc did not improve from 0.73876.

There are a total of 100 *epochs.* They stopped training at 15 to 18th *epochs*. This is because of the function EarlyStopping that helps to avoid the problem of overfitting.

The accuracy remains constant after the 15th *epochs,* whereas, val_acc starts decreasing due to which training stops.

*val_acc*:- It is the measure of how good are the predictions of the model. So, in our case, the model is very well trained till the 15th *epoch*, and after that, the training is not necessary.

acc:- It gives the percentage of instances that are correctly classified. In our case, the percentage is 93.15%.

*val_loss*:- val_loss is the loss that is applied to the testing set, whereas, the *loss* is applied to the training set. *val_loss* is the best to depend on to avoid the problem of overfitting. Overfitting is when the model is fit too closely to the training data.

**Figure 11:**
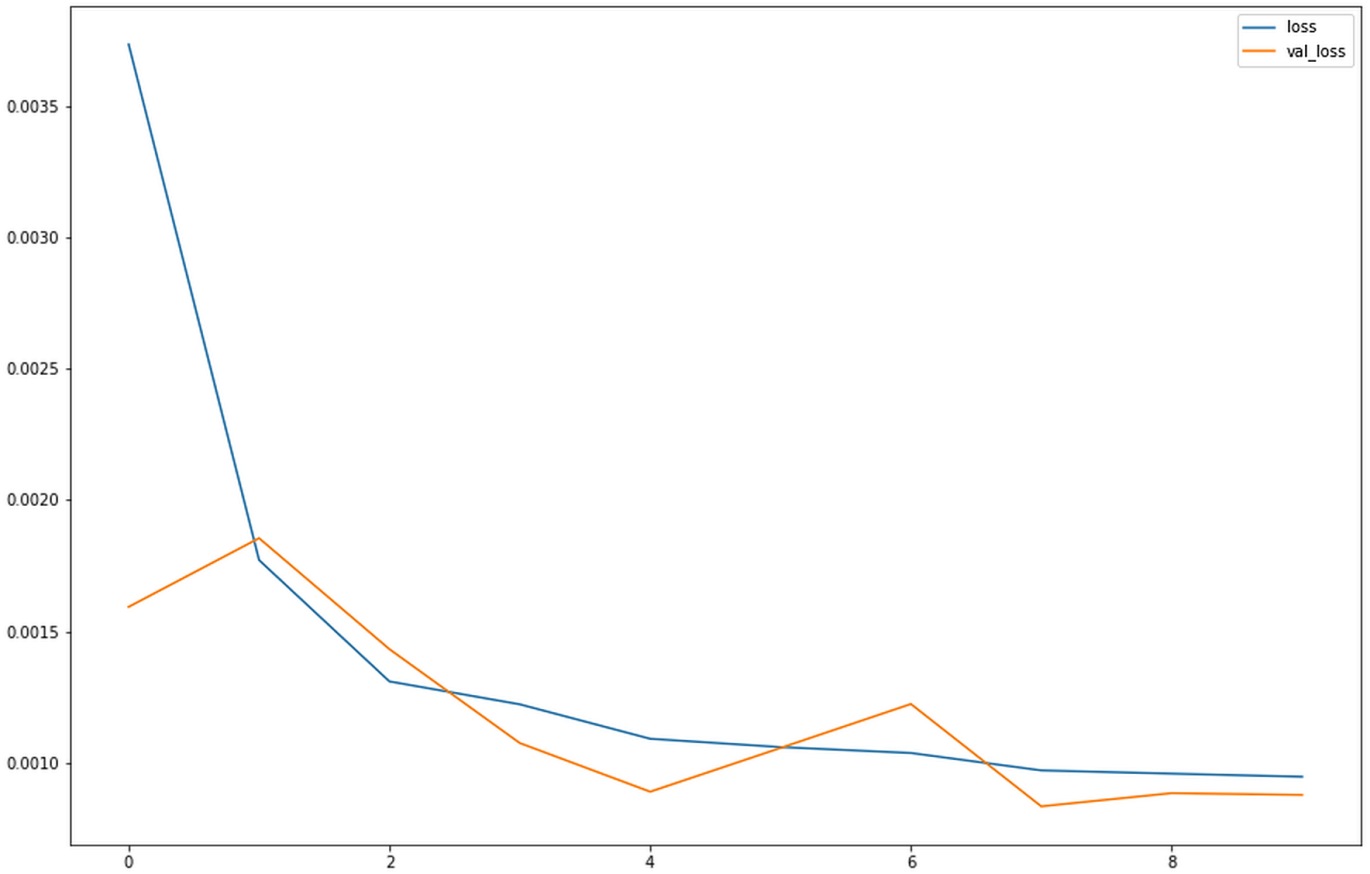
Loss for LSTM for OHLC Data. Graph comparing the loss vs. val_loss for the OHLC dataset for the LSTM model shows as the number of epochs progresses, the values of loss and val_loss tend to decrease and plateau to approximately 0.0010.

The *val_loss* and *loss* keep on decreasing, whereas acc and *val_acc* are kept on increasing this shows that our model is well-trained. Figure 11 visualises the loss over number of epochs.

**Figure 12:**
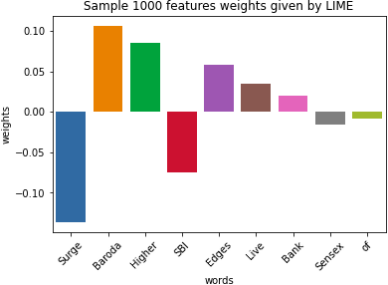
Sample 1,000 feature weights given by LIME. Result obtained by LIME tool after modeling it. We get a cumulative 1,000 explanation features. Each word is weighted.

As according to the plot obtained after training, we observe that initially, the *loss* is very high; however the *validation loss* is low relatively. Once the *epochs* progress, the *loss* decreases, thus providing better learning for our model. The point where the loss and *validation* loss plateaus are the point where no further learning might be possible with the same amount of data for the same number of *epochs.* Although the accuracy of LSTM-CNN with news headlines data is less than LSTM with OHLC data, since we are interpreting the model, it is adding another perspective of viewing at the model, which makes it a better model. When compared with RNN-CNN (Tan et al., 2019; Zhang, Chen & Huang, 2018) model , which it performs way better since not only does it remember small term information but also long term information.

### Results of XAI

As for the first model,as shown in Fig. 12 , we get a cumulative 1,000 explanation features. The word surge is mostly related in a negative context and hence is having a negative weight. Similarly, higher is mostly in those sentences which depict the growth of stocks and hence is having positive weight. We can also observe the word Sensex having near about neutral weight as it has both positive and negative references. Thus depicts both falls as well as the rise of stocks. By Table 4, we can thus interpret our data and thus understand the biases in the dataset. Our model is not just a black box now, and the customers know the insides of the system through just one graph. Thus we achieve the explanations along with the insights from the data.

**Table 4:** Results of LIME tool. The sample of the result obtained by the lime tool where each word is assigned weight or bias.

Test Words	Weights or biases (approx)
Surge	−0.15
Baroda	+0.1
Higher	+0.8
Edges	+0.5
Live	+0.3
Bank	+0.2
Sensex	−0.1
Of	−0.05
SBI	−0.7

### Test results

**Figure 13:**
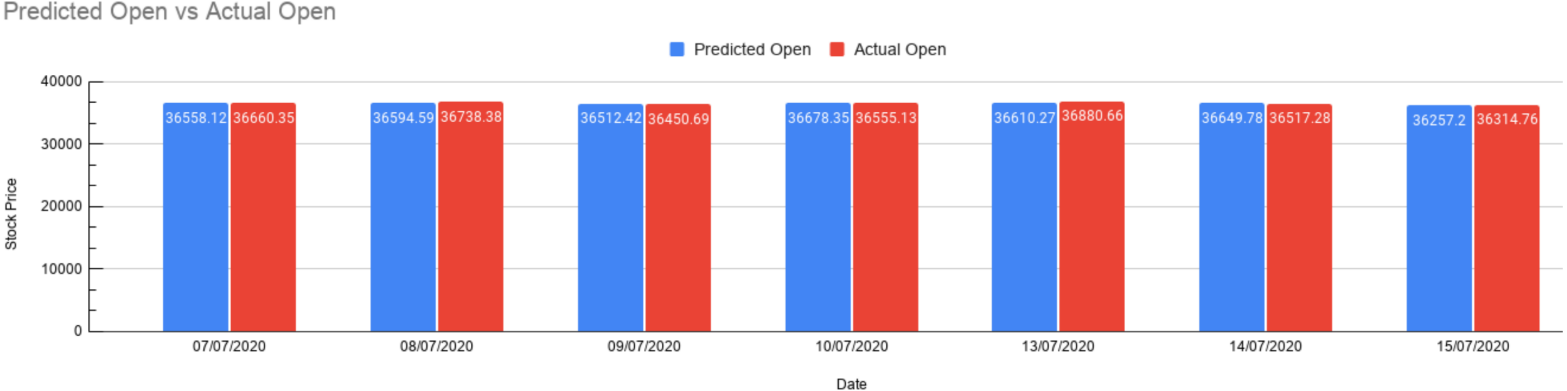
Prediction results. Bar graph depicting Predicted Open vs. Actual Open. The red bar shows the Actual Open Prices, and the blue bar shows the predicted opening prices for 7 days.

As shown in above Fig. 13, the trained model can now be tested for predictions. The blue bar represents the predicted value, and the red bar represents the actual value obtained from the dataset. This gives us a fair understanding of the output. If we want to test for the real-time news, we can enter news that gets preprocessed and get a measure of the opening price, i.e., how much did the opening price rise or fall from the previous opening price. We present values in Table 4 for getting the exact idea of these values and gauge the accuracy of our predictions.

 The table below shows the following: date of actual open , previous closing price, predicted opening price by our model, actual opening price, rise / fall in price, error percentage, chi-squared test.

Here the:

Rise/fall is given by: (Predicted Open -Previous Close) (1.23)

Error percentage is given by: abs((Actual-predicted)/(Actual ))*100 (1.24)

Chi-square is given by:(actual - predicted)^2^/predicted (1.25)

As presented above in Table 5, seven test cases have been stated with the stock market prices along with the rise and fall of those values. The error represents the value difference in percentage (Actual-predicted by our model) and for our test cases, it’s showing in the range 0.15 to 0.73. Thus, we could manage to predict the Indian stock market price using XAI by referring to the impact of financial news articles. Also the chi-square test allows us to assess how much is the observed data varying from actual data.

**Table 5:** Set of predictions made. The table with the final set of predictions along with comparisons with Actual Opening Prices, calculations for Rise/Fall, Error Percentage, and the chi-squared test.

**Test case**	**Date**	**Previous close**	**Predicted open**	**Actual open**	**Rise/fall**	**Error(%)**	**Chi-squared test**
1	07∕07∕2020	36487.28	36558.12	36660.35	170.84	0.27	0.28
2	08∕07∕2020	36674.52	36594.59	36738.38	−79.93	0.39	0.56
3	09∕07∕2020	36329.01	36512.42	36450.69	183.41	0.16	1.52
4	10∕07∕2020	36737.69	36678.35	36555.13	−59.34	0.33	0.41
5	13∕07∕2020	36594.33	36610.27	36,880.66	15.94	0.73	1.98
6	14∕07∕2020	36693.69	36649.78	36,517.28	−43.91	0.21	0.48
7	15∕07∕2020	36033.06	36257.20	36,314.76	224.14	0.15	0.01

## Conclusion and Future Scope

The financial news articles play a major role in the movement of the stock price. Financial news plays a dominant factor in how a particular company’s stock is perceived by the investors at a given time. Making predictions based on news headlines can help budding investors learn how and when stock prices fall or rise and take a decision based on the same. Our proposed model created an explainable model that gives this explanation as well as maintains and thus makes the output meaningful. This was done with the XAI using the tool LIME. Future research directions could be automated predictions from a news headline from a financial website and a multilingual financial news headline prediction. We can also add emotion-based GIFs to add a fun element and make it more appealing for the learner. The prediction model can be used as a decision-maker for an algorithmic trading model.

##  Supplemental Information

10.7717/peerj-cs.340/supp-1 Supplemental Information 1 Pulse Dataset

## Additional Information and Declarations

### Data Availability

The following information was supplied regarding data availability: Raw data, including financial news headlines obtained from Pulse and data from Yahoo Finance, is available in the Supplementary Files. Code is available at GitHub: https://github.com/Hrituja/Stock-Market-Prediction.
